# Why we need a national repository of consumer product lead surveillance data

**DOI:** 10.1038/s41370-023-00525-1

**Published:** 2023-02-07

**Authors:** Kolapo Alex-Oni, Slavenka Sedlar, Paromita Hore

**Affiliations:** Bureau of Environmental Disease and Injury Prevention, New York City Department of Health and Mental Hygiene, 125 Worth Street, CN34C New York, NY USA

*“Is this a problem in the rest of the United States?”* was a question posed by delegates from an international governmental agency during a meeting convened by the New York City (NYC) Department of Health and Mental Hygiene (DOHMH). The meeting was called to discuss the issue of elevated levels of lead in spices that were purchased in the representatives’ country and associated with lead poisonings in NYC residents. “*Is the issue of lead in spices seen in other jurisdictions across the United States?”* asked the delegates. *“If so, where are the supporting data*?” While some research studies and case reports affirm that NYC is not the only jurisdiction contending with exposures to lead-containing consumer products [1–7], these types of findings may not always be reported or are released ad hoc and thus, cannot be sufficiently harnessed to show the potential depth of the issue. A comprehensive and systematically tracked national dataset could be an effective solution to address this need. Such a centralized repository of consumer product lead surveillance data could reveal the extent of product-related lead exposures around the United States and help to monitor associated trends. This data can also illuminate product use patterns among various demographics and inform intervention strategies to reduce potential lead exposures.

Despite declines in blood lead levels of children and adults in the United States, lead exposures, including from lead-containing products [8], still occur, and because of the many harmful health effects of lead exposure [9] —often in the absence of symptoms—prevention is key. Many factors are increasing the need for systematic monitoring of product-related lead exposures: (1) the growth of immigrant and refugee populations, who are disproportionately impacted by lead poisoning—partly due to their use of lead-containing cultural products [10], (2) the proliferation of cross-cultural consumption of traditional foods, health remedies and similar products [11] and (3) the interconnectedness of the global supply chain.

Such a surveillance system has been in-place in NYC for over a decade. DOHMH receives blood lead test results for NYC residents and routinely investigates child and adult lead poisoning cases [1]. During case investigations, DOHMH uses a detailed risk assessment questionnaire and conducts environmental sampling, when pertinent, to identify potential lead sources. Samples of products suspected to contain lead and that may be mouthed or ingested are collected by DOHMH and tested for lead by an accredited laboratory. Laboratory results for each sample collected, along with a description of the sample, including product name, usage and purchase source information are documented electronically in a proprietary Structured Query Language Server database.

Through these investigations, DOHMH has identified a variety of lead-containing consumer products from around the world, such as certain foods, spices, health remedies, traditional cook or dish ware and cultural powders, used by diverse groups, including NYC’s South Asian, Georgian and Mexican communities (Fig. 1) [12–16]. Lead-containing consumer products are the second most common potential source of lead exposure for NYC children; additionally, in approximately, 80% of NYC pregnant cases, imported products are a potential lead source [17].

**Fig. 1:**
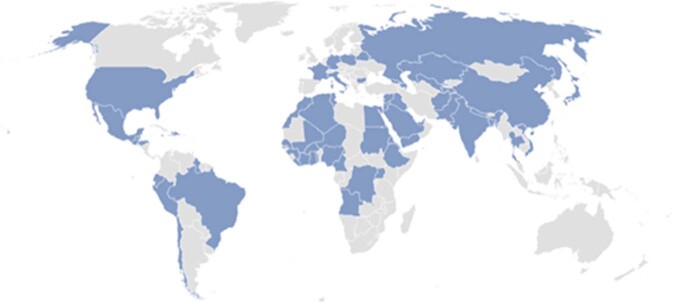
Countries of purchase for products with detectable lead identified during NYC investigations, 2010-2022. Blue shading indicates countries where products with detectable lead levels were purchased, illustrating the global availability of lead-containing products.

DOHMH’s systematic monitoring of consumer product lead data has not only helped to identify new lead sources impacting New Yorkers but has also informed risk communication strategies for communities at an increased risk for lead poisoning due to use of such products [18]. In addition, the identification of these types of products has driven enforcement activities to eliminate their availability in the local marketplace, resulting in the removal of tens of thousands of hazardous consumer products from NYC store shelves. Broader impact of the NYC data is evidenced by surveillance and enforcement by external agencies, product recalls, import alerts, national and international health alerts and investigative activities in the products’ countries of origin (Fig. 2). Although the NYC consumer product lead surveillance dataset is robust, this data only provides a local perspective.

**Fig. 2:**
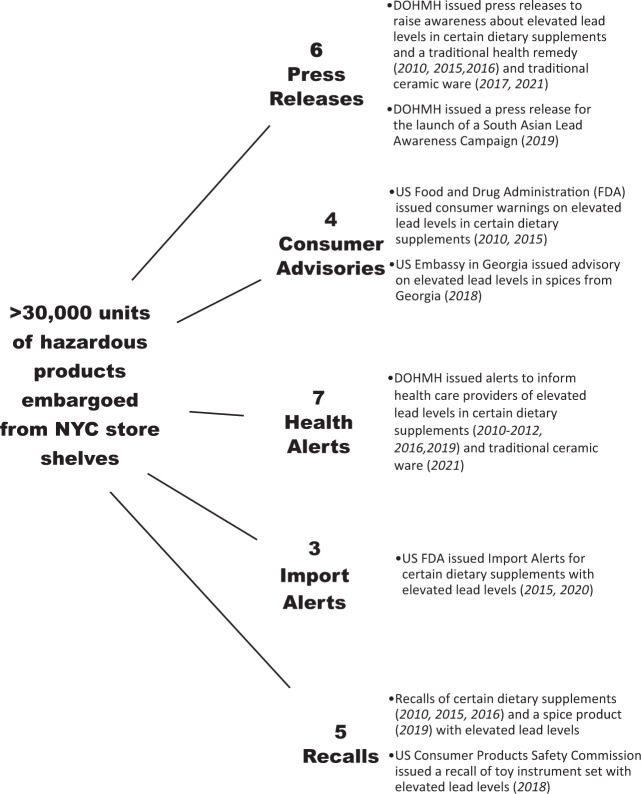
Selected actions triggered by NYC DOHMH consumer product surveillance, 2010-2022. NYC DOHMH uses a multi-pronged approach that incorporates enforcement and education activities to address lead-containing consumer products. NYC product surveillance has resulted in the removal of tens of thousands of hazardous consumer products from NYC stores and the issuance of press releases, consumer advisories, health alerts, import alerts and recalls. Some of these actions are illustrated in this figure.

## A national data repository can facilitate a solution

An effort to track national lead data for the purpose of identifying populations at risk is not entirely new. The Centers for Disease Control and Prevention’s Adult Blood Lead Epidemiology and Surveillance (ABLES) program has a similar purpose. ABLES collects blood lead level data from state programs to examine trends and guide interventions to prevent work-related lead exposures. As a result of this initiative, ABLES data has been used to monitor workplace lead exposure trends, initiate investigations and promote prevention interventions [19].

A similar national surveillance system could be implemented for consumer product data collected during local and state lead poisoning investigations, whereby product-related information, such as product name, purchase source, usage patterns, supply chain details and lead concentration, along with associated blood lead levels, could be systematically reported. Since lead poisoning prevention programs across the country currently investigate cases of elevated blood lead levels to identify potential lead exposures, an opportunity to track non-paint lead sources already exists. Such a national repository of consumer product lead data could elucidate and quantify the extent of product-related lead exposures around the country, identify patterns of exposure and motivate data-driven solutions to address these types of exposures. Such evidentiary data can also inform policies aimed at removing lead sources at the countries of origin—protecting consumers nationally and internationally—by engaging global stakeholders, including international health authorities, non-governmental organizations and academics.

The overall benefits of a national repository of consumer product lead surveillance data are evident and wide-ranging, with implications for case management, education, outreach and policy development. However, the groundwork must be laid federally to make this process standardized and effective, and ultimately, to be able to assess the issue on a national scale. Without a national dataset of lead-containing consumer products associated with elevated blood lead levels, an understanding of the magnitude of this issue will remain fragmented, leaving us to wonder, “*Is this a problem in the rest of the United States?”*.

## Data Availability

Data generated and analyzed in support of this comment article is available on request from the corresponding authors.
